# Comprehensive immune profiling and immune-monitoring using body fluid of patients with metastatic gastric cancer

**DOI:** 10.1186/s40425-019-0708-8

**Published:** 2019-10-21

**Authors:** Hyung Soon Park, Woo Sun Kwon, Sejung Park, Eunji Jo, So Jung Lim, Choong-kun Lee, Jii Bum Lee, Minkyu Jung, Hyo Song Kim, Seung-Hoon Beom, Jun Yong Park, Tae Soo Kim, Hyun Cheol Chung, Sun Young Rha

**Affiliations:** 1 Division of Medical Oncology, Department of Internal Medicine, St. Vincent’s Hospital, The Catholic University of Korea, Suwon, South Korea; 2 Songdang Institute for Cancer Research, Yonsei University College of Medicine, Seoul, South Korea; 3 Brain Korea 21 Project for Medical Sciences, Yonsei University College of Medicine, Seoul, South Korea; 4 Division of Medical Oncology, Department of Internal Medicine, Yonsei Cancer Center, Yonsei University College of Medicine, 50-1 Yonsei-Ro, Seodaemun-gu, Seoul, 120-752 South Korea; 5 Division of Gastroenterology, Department of Internal Medicine, Yonsei University College of Medicine, Seoul, South Korea

**Keywords:** Cytokines, Immune profile, Body fluid, Metastatic gastric cancer

## Abstract

**Background:**

The aim of this study is to profile the cytokines and immune cells of body fluid from metastatic gastric cancer (mGC), and evaluate the potential role as a prognostic factor and the feasibility as a predictive biomarker or monitoring source for immune checkpoint inhibitor.

**Methods:**

Body fluid including ascites and pleural fluid were obtained from 55 mGC patients and 24 matched blood. VEGF-A, IL-10, and TGF-β1 were measured and immune cells were profiled by fluorescence assisted cell sorting (FACS).

**Results:**

VEGF-A and IL-10 were significantly higher in body fluid than in plasma of mGC. Proportion of T lymphocytes with CD69 or PD-1, memory T cell marked with CD45RO, and number of Foxp3+ T regulatory cells (Tregs) were significantly higher in body fluid than those in blood of mGC. Proportion of CD8 T lymphocyte with memory marker (CD45RO) and activation marker (HLA-DR), CD3 T lymphocyte with PD-1, and number of FoxP3+ Tregs were identified as independent prognostic factors. When patients were classified by molecular subgroups of primary tumor, VEGF-A was significantly higher in genomically stable (GS)-like group than that in chromosomal instability (CIN)-like group while PD-L1 positive tumor cells (%) showed opposite results. Monitoring immune dynamics using body fluid was also feasible. Early activated T cell marked with CD25 was significantly increased in chemotherapy treated group.

**Conclusions:**

By analyzing cytokines and proportion of immune cells in body fluid, prognosis of patients with mGC can be predicted. Immune monitoring using body fluid may provide more effective treatment for patients with mGC.

**Electronic supplementary material:**

The online version of this article (10.1186/s40425-019-0708-8) contains supplementary material, which is available to authorized users.

## Background

Gastric cancer ranks the fourth among the most common cancers worldwide and the third in mortality rate [1]. It is the second most common cancer in Korea. About 30,000 new cases are diagnosed in 1 year [2]. About 30~35% of gastric cancer patients show initial distant metastasis. Palliative chemotherapy is a standard treatment [3]. The survival of patients with metastatic gastric cancer (mGC) is less than 2 years. Anti-HER2 therapy with trastuzuamb can prolong the survival of HER2 positive patients up to 13 months [4, 5]. After the ToGA (Trastuzumab for Gastric Cancer) study, the first positive study in advanced gastric cancer using target agent, many studies have attempted to find targeted therapy according to molecular aberrations found in gastric cancer. Such studies are supported by genetic profiling of tumor in several groups including The Cancer Genome Atlas Research Network (TCGA) [6]. After failure of several kinds of targeted agents, immune checkpoint inhibitor (ICI) has emerged as a new treatment option for gastric cancer. Nivolumab and pembrolizumab have shown promising antitumor activity [7, 8]. The biomarker of ICI is a major issue in cancer field including metastatic gastric cancer. It might help us better indicate which patients are most likely to have benefits [7, 9]. Several biomarkers such as tumor mutation burden and programmed death-ligand 1 (PD-L1) status by immunohistochemistry (IHC) have been suggested. However, none of them is considered as a standard biomarker. The role of immune cells in immune checkpoint inhibitor response is well known, especially tumor infiltrating lymphocytes (TIL) in tumor microenvironment. Thus, tumor can be categorized as inflamed or non-inflamed. Inflamed tumor is characterized by the presence of TIL, high density of CD8^+^ T cell, and expression of PD-L1 in tumor or immune cells. Collective clinical evidence suggests that ICI is more effective for inflamed tumors [10].

However, not all patients with inflamed tumors respond to ICI. T cell receptor (TCR)-peptide-major histocompatibility complex (pMHC) binding is the central event in the activation of T cell. Activation antigens on T cell include CD25, CD26, CD38, CD54, CD69, and HLA-DR [11, 12]. Activated T cells can trigger TME evolution including up-regulation of inflammatory/suppressive cytokines, immune-inhibitory cellular recruitment, and aberrant tumor vasculature related to innate/acquired resistance. The complex crosstalk among cancer cells, immune cells, and tumor microenvironment is closely connected to each other. Activation antigens on T cells could be detected according to time. CD69 and CD25 are early antigens whereas HLA-DR is a late marker [11]. The kinetics of expression of early activation markers (CD69 and CD25) was similar to that of PD-1 expression [13]. In tumor tissue, chronic exposure to antigen and the development of dysfunctional or exhausted effector T cell are accompanied by high expression of one or more inhibitory receptors including PD-1, lymphocyte activation gene 3 (LAG-3), and T-cell immunoglobulin and mucin-domain containing-3 (TIM-3) [14]. In addition, regulatory T cell and memory T cell play a role in the control of tumor growth and progression.

About 30–40% of mGC patients have malignant ascites associated with peritoneal carcinomatosis [15, 16]. Ascites has a different tumor microenvironment from primary tumor, and it has high levels of immune suppressive cytokines and immune cells [17–19]. Especially, several cytokines such as VEGF-A, IL-10, and TGF-β1 have immune suppressive function [20]. They are directly or indirectly related to angiogenesis which has close interaction with immune cells for immune surveillance [21, 22]. The role and profile of these cytokines have been mostly studied in ovarian cancer. Their expression level can affect patient prognosis and drug resistance [23]. However, little is known cytokines and immune signature in malignant ascites of gastric cancer. Only a small number of studies have dealt with this subject [20]. Ascites reflects tumor microenvironment. It has advantage such as easier acquisition than tumor biopsy. Malignant ascites contains several kinds of cytokines and plenty of immune cells having directly contact with tumor cells, suggesting that malignant ascites might serve as a good resource for immune monitoring of mGC patients. Therefore, the aim of this study was to obtain profiles of cytokines and immune cells of body fluid including ascites and pleural fluid in mGC and evaluate their potential roles as prognostic factors. The feasibility of using ascites as a predictive biomarker and a monitoring source for immune checkpoint inhibitor was also examined.

## Methods

### Study population

Between December 2014 and April 2016 at Yonsei Cancer Center, Seoul, Korea, body fluid including ascites and pleural fluid were obtained in a prospective, nonselective fashion from 55 mGC patients via paracentesis or catheter drainage. Twenty-four matched blood samples were also taken before or at the time of body fluid acquisition. Cancer cells from body fluid were confirmed by pathologist using cell block. Eleven non-cancerous ascites and 4 matched blood samples were obtained from patients with Child-Pugh B/C liver cirrhosis. Blood samples from 15 healthy volunteers were used as controls. Clinical data including age and sex were collected for all study subjects. The following clinico-pathologic information was obtained by reviewing electronic medical records (EMR) of gastric cancer (GC) patients: disease presentation (recurrent or metastatic), type of surgery, differentiation, Lauren classification, HER2 status, type of body fluid, acquisition timing of body fluid, and survival time. This study was approved by the Institutional Review Board of Severance Hospital (No. 4–2014-0638).

### Measurement of immune suppressive cytokine

VEGF-A, IL-10, and TGF-β1 which are known immune suppressive cytokines were selected for measurement to evaluate their clinical roles and associations with immune cells. Plasma was isolated from collected 10 ml peripheral blood in EDTA tube using Ficoll-Paque™ PLUS (GE Healthcare, Sweden) gradient centrifugation and stored at − 80 °C until assay. Body fluid was incubated in 10 X RBC lysis buffer (Biolegend, CA, USA) to remove RBC and centrifuged (5 min, 1500 rpm). The supernatant was separated, aliquoted, and stored at − 80 °C until analysis. Plasma and body fluid supernatant were used to measure levels of circulating VEGF-A, IL-10, and TGF-β1 using a commercially available enzyme-linked immunosorbent assay (ELISA) kit (Quantikine; R&D Systems Abingdon, UK) following the manufacturer’s protocols. Samples were measured in duplicates and mean value was presented as final concentration. ELISA plates were read on a Sunrise absorbance microplate reader (TECAN, Switzerland).

### Isolation of PBMC and lymphocyte in malignant fluid

Peripheral blood mononuclear cells (PBMCs) were isolated from 10 ml peripheral blood collected into EDTA tube using Ficoll-Paque™ PLUS (GE Healthcare, Sweden) gradient centrifugation. Tumor associated lymphocytes (TALs) from 100 to 500 mL malignant fluid were isolated using the following protocol. Briefly, the fluid was incubated with 10 X RBC lysis buffer (Biolegend, CA, USA) to remove RBC and centrifuged in 50 ml tubes at 1500 rpm for 5 min. After two washes with PBS (phosphate buffered saline), isolated cells were suspended in 1 ml of Cellbanker-2 (ZENOAQ, Japan) and stored at − 80 °C until analysis.

### Flow cytometry and antibodies

Flow cytometry was performed using FACS LSR2 (BD Biosciences, CA, USA). Data were analyzed using FlowJo software (FlowJo, LLC, OR, USA). Fluorescence-conjugated monoclonal antibodies were purchased from the following sources: Human LAG-3 Alexa Fluor® 488-conjugated Antibody (R&D system, MN, USA); PE/Cy7 anti-CD3, FITC anti-CD4, PE anti-CD8, FITC anti-CD45RO, FITC anti-HLA-DR, APC anti-CD25, APC/Cy7 anti-CD69, APC anti-human CD279 (PD-1), APC/Cy7 anti-human CD366 (Tim-3), and PE anti-FoxP3 (BioLegend, CA, USA). Furthermore, cells were stained with PE anti-human CD274 (B7-H1, PD-L1, BioLegend, CA, USA) to identify proportion of PD-L1 positive tumor cells in body fluid.

For Treg cell staining, cells were stained with various antibodies except FoxP3 antibody for which cells were fixed and permeabilized with eBioscience™ FoxP3 Fixation/Permeabilization solution (Thermo Fisher Scientific, MA, USA). FoxP3 antibodies were administered after permeabilization for intracellular staining of Tregs. FACS analyses were performed for cells isolated from malignant fluid and peripheral blood. First, levels of CD4+ and CD25+ T cells in cells isolated from these two sources (malignant fluid and peripheral blood) were measured. Next, we quantified the percentage of cells that were positive for FoxP3 in the CD4 + CD25+ T cell population.

### Molecular subtype of primary tumor by histochemistry

Gastric cancer panel practically used in our institution has 10 markers, including Epstein-Barr virus encoded small RNAs (EBER) in-situ hybridization, mismatch repair proteins (MLH1, PMS2, MSH2, and MSH6), receptor tyrosine kinases (RTKs; HER2, EGFR, and MET), PTEN, and p53 protein expression by IHC using formalin-fixed, paraffin-embedded (FFPE) tissue blocks from paired primary stomach cancer. Detailed methods of in situ hybridization (ISH) and IHC staining have been described in our previous study [24]. Patients were categorized by The Cancer Genome Atlas (TCGA) molecular subtypes, including Epstein Barr Virus (EBV) positive, microsatellite instability (MSI), genomically stable (GS)-like, and chromosomal instability (CIN)-like. CIN-like group patients had overexpression of HER2 (ISH 3+ or ISH2 + with amplification by ISH), EGFR (2+ or 3+), MET (2+ or 3+), and PTEN loss. Others with all markers negative were included in the GS-like group. In landscape analysis, continuous variables were expressed by categorized value (low vs. high group) which was determined by the best cut off point.

### Statistical analysis

Differences in cytokine and immune profiles among blood and body fluid samples were compared using the Mann-Whitney U test. Pearson’s correlation coefficient was calculated to determine relationships among variables. A value of higher than 0.7 means highly positive correlation [25]. Continuous variable were transformed into categorical variables with high or low to calculate the maximizing hazard ratio (HR) based on log-rank statistics presented by Contal and O’Quigley [26]. The overall survival (OS) was defined as the time from body fluid acquisition to death from any cause. Time to event endpoint was analyzed by Kaplan-Meier survival curves using the log-rank test. Scoring system using ascites cytokines according to best-cut off point was made to establish a prognostic model. A number of cytokines with high expression levels including VEGF-A, IL-10, and TGF-β1 were categorized as 0–1 and 2–3. To determine independent prognostic factor, significant prognostic factors identified in univariate analysis were analyzed with multivariate Cox proportional hazard model using forward stepwise analysis. A *p-*value of less than 0.05 was considered statistically significant. PASW Statistics 18.0 (SPSS Inc. Chicago, IL, USA) was used for all statistical analyses.

## Results

### Patient characteristics

Baseline characteristics of enrolled patients are shown in Table 1. The median age of 15 healthy volunteers was 63 years (range, 27–77 years) and eight (53%) were females. Eleven Child-Pugh B/C liver cirrhosis patients with ascites were enrolled as controls. Their median age was 53 years (range, 35–79 years) and seven (63.6%) were males. Fifty-five mGC patients with ascites or pleural fluid were enrolled. Their median age was 58 years (range, 25–75). There were 66% males. Fourteen (25%) patients had recurrent gastric cancer. Thirty-nine (71%) patients had poorly differentiated or signet ring cell features. Eight (14.5%) patients were HER2 positive. Forty-four (80%) patients had palliative chemotherapy history at the time of body fluid acquisition.

**Table 1:** Baseline characteristics

		No.	%
Healthy volunteers	(*n* = 15)		
Age	median, range	63	27-77
Sex	Male	7	46.7
	Female	8	53.3
Non-cancerous patients	(*n* = 11)		
Age	median, range	53	35-79
Sex	Male	7	63.6
	Female	4	36.4
Gastric cancer patients	(*n* = 55)		
Age	median, range	58	25-75
Sex	Male	36	65.5
	Female	19	34.5
Disease presentation	Recurrent	14	25.5
	Metastatic	41	74.5
Type of surgery	Total gastrectomy	9	16.4
	Subtotal gastrectomy	10	18.2
	No	36	65.5
Differentiation	WD	3	5.5
	MD	9	16.4
	PD	27	49.1
	SRC	12	21.8
	Others^a^	4	7.3
Lauren	Intestinal	7	12.7
	Diffuse	10	18.2
	Mixed	1	1.8
	Unknown	37	67.3
HER2	Negative	46	83.6
	Positive	8	14.5
	Unknown	1	1.8
Type of body fluid	Ascites	46	83.6
	Pleural fluid	9	16.4
Previous palliative chemotherapy at acquisition of body fluid	Chemotherapy-naïve	11	20.0
	1	14	25.5
	2	15	27.3
	≥3	15	27.3

*WD* Well differentiated, *MD* Moderate differentiated, *PD* Poorly differentiated, *SRC* Signet ring cell carcinoma, *No* Number

Others^a^: mucinous adenocarcinoma, undifferentiated carcinoma

### Comparison of immune suppressive cytokines in healthy volunteers, liver cirrhosis patients, and gastric cancer patients

**Fig. 1:**
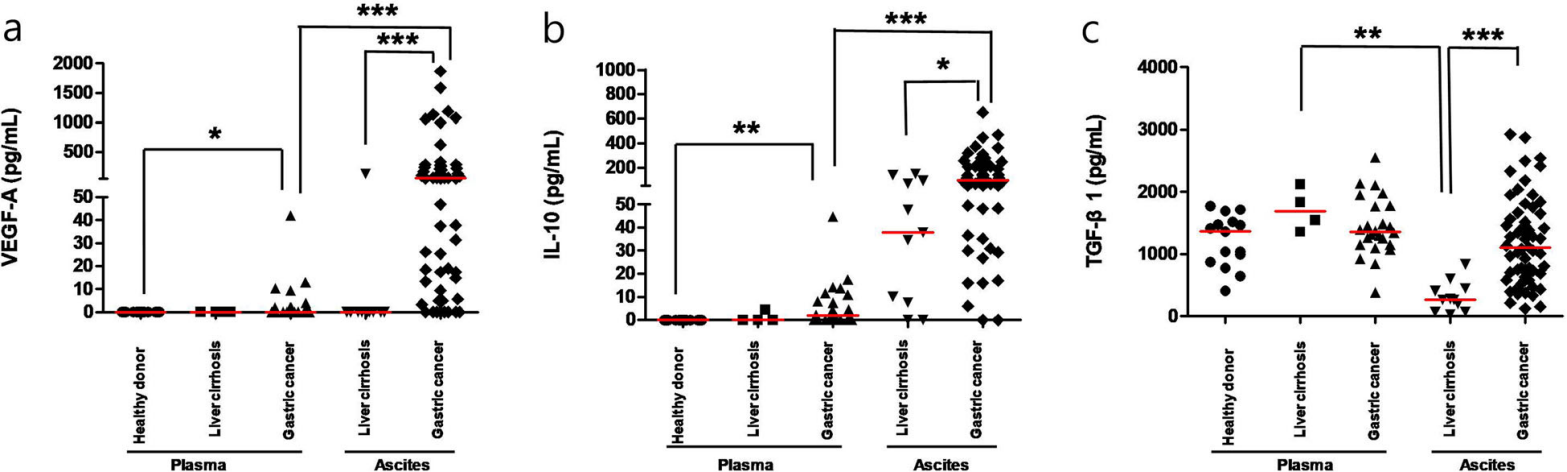
Pro-angiogenic, immune modulatory cytokine, and immunosuppressive cytokines have different patterns in plasma and body fluid among healthy volunteer, non-cancerous patients, and gastric cancer patients. **a** Plasma VEGF-A in mGC were significantly higher than those in healthy volunteers (*P* = 0.013). VEGF-A levels in mGC body fluid were significantly higher than those in mGC plasma and non-cancerous body fluid. **b** Plasma IL-10 levels in mGC were significantly higher than those in healthy volunteers (*P* = 0.001). IL-10 levels in mGC body fluid were significantly higher than those in mGC plasma and non-cancerous body fluid (*P* = 0.014). **c** TGF-β1 levels in non-cancerous body fluid were significantly lower than those in non-cancerous plasma (*P* = 0.005) and mGC body fluid. mGC, metastatic gastric cancer. Red line indicates median value. Mann-Whitney U test was used for statistical analysis. *P < 0.05, **P < 0.01 ***P < 0.001

Median values of plasma and body fluid cytokines are shown in Additional file 1: Table S1. Plasma VEGF-A and IL-10 levels in mGC were significantly higher than those in healthy volunteers (*P* = 0.013 and *P* = 0.001, respectively). VEGF-A and IL-10 levels in mGC body fluid were significantly higher than those in mGC plasma and non-cancerous body fluid (Fig. 1a-b). In contrast, ascites TGF-β1 levels in liver cirrhosis samples were lower than those in other samples, and remaining samples showed no significant difference in TGF-β1 level among each other (Fig. 1c). When correlations of cytokines between plasma and body fluid in mGC were analyzed, levels of VEGF-A, but not those of IL-10 or TGF-β1, showed significant correlations (*P* = 0.004, correlation coefficient, r = 0.5647) (Additional file 4: Figure S1). In addition, there were no significant correlations among cytokines in body fluid (Additional file 5: Figure S2).

### Immune cell profiling of paired PBMC and body fluid in mGC

**Fig. 2:**
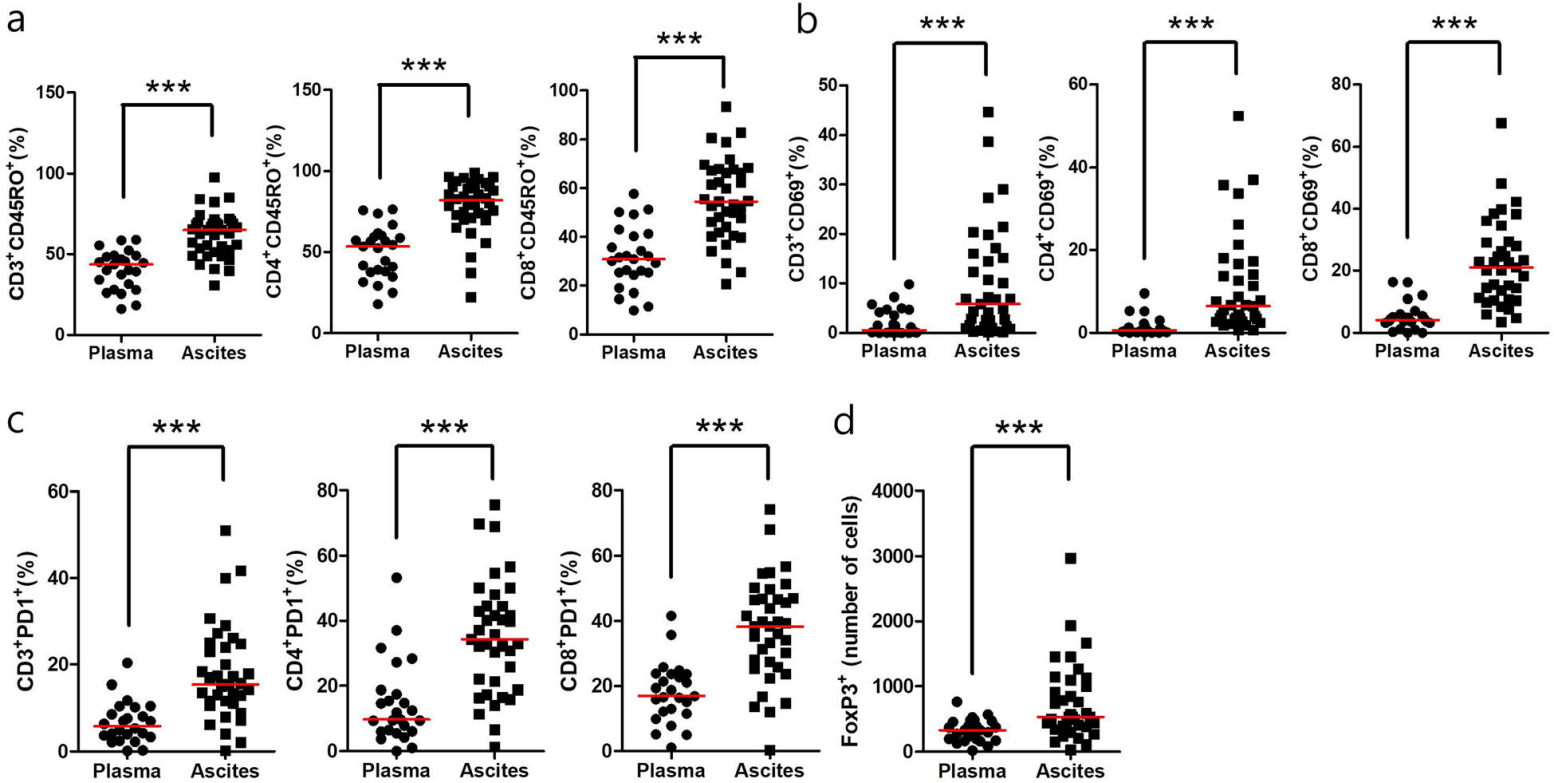
Proportion of immune cells were significantly higher in body fluid than those in peripheral blood. **a** Immune cell profiling of mGC body fluid by FACS analysis were compared to those of mGC peripheral blood. Proportion of memory T cell marked with CD45RO (CD3CD45RO, CD4CD45RO and CD8CD45RO) were significantly higher in body fluid than those in peripheral blood of mGC. **b** Activated T lymphocytes (early activation marker CD3CD69, CD4CD69, CD8CD69) were significantly higher in body fluid than those in peripheral blood of mGC. **c** T lymphocytes with inhibitory marker including PD-1 (CD3PD1, CD4PD1 and CD8PD1) were significantly higher in body fluid than those in peripheral blood. **d** Number of FoxP3+ T regulatory cells (Tregs) were significantly higher in body fluid than those in peripheral blood. mGC, metastatic gastric cancer. Red line indicates median value. Mann-Whitney U test was used for statistical analysis. *P < 0.05, **P < 0.01 ***P < 0.001

Results of immune cell profiling of peripheral blood and body fluid in mGC are summarized in Additional file 2: Table S2. Proportion of CD8/CD3 T cells showed a higher tendency while CD4/CD8 ratio showed a lower tendency in body fluid than those in peripheral blood (*P* = 0.073 and *P* = 0.075, respectively) of mGC. Proportion of memory T cell marked with CD45RO (CD3CD45RO, CD4CD45RO and CD8CD45RO) and activated T lymphocytes (early activation marker CD3CD69, CD4CD69, CD8CD69; late activation marker CD4HLA-DR) were significantly higher in body fluid than those in peripheral blood of mGC (Fig. 2a, b). In addition, T lymphocytes with inhibitory marker including PD-1 (CD3PD1, CD4PD1 and CD8PD1) and number of FoxP3+ T regulatory cells (Tregs) were significantly higher in body fluid than those in peripheral blood (Fig. 2c-d). Higher number of FoxP3+ Tregs in body fluid was significantly associated with increased T cell with inhibitor marker such as LAG3 and TIM3 (Additional file 6: Figure S3).

We then compared levels of cytokines and immune cell profiling. VEGF-A and proportion of T cells with CD69 or CD25 showed significant positive correlations (CD3CD69, correlation coefficient, r = 0.377, *P* = 0.021; CD4CD69, r = 0.374, *P* = 0.023; CD4CD25, r = 0.357, *P* = 0.03; CD8CD25, r = 0.688, *P* < 0.001). In contrast, VEGF-A and number of FoxP3+ Tregs showed significant negative correlation (r = − 0.339, *P* = 0.043) (Additional file 7: Figure S4), suggesting that increased VEGF-A level might be related to immune suppressive microenvironment.

### Survival analysis for immune monitoring as a prognostic factor

**Fig. 3:**
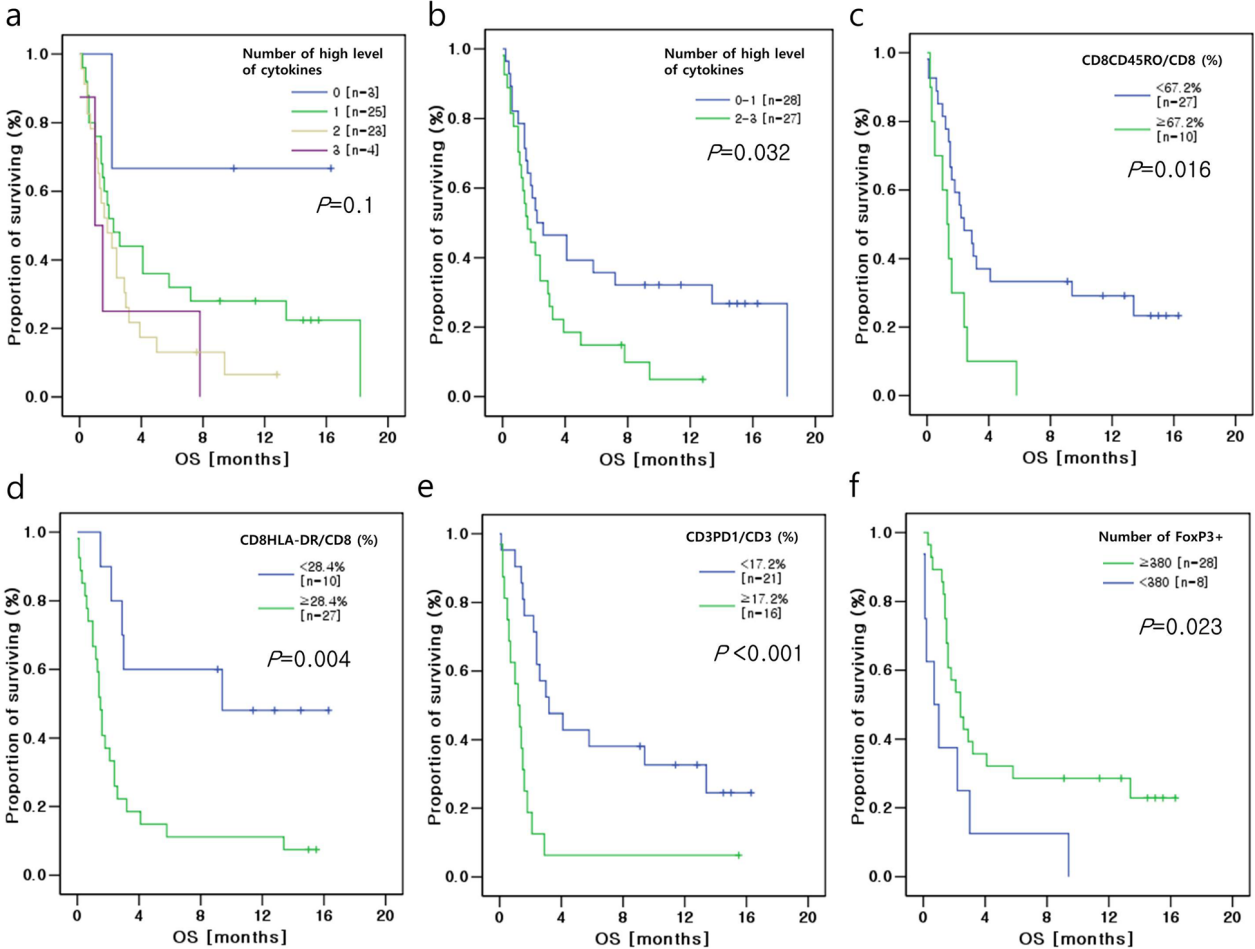
Immune cytokines scoring model (patients were divided into two groups by number of high levels of three cytokines - VEGF-A, IL-10 and TGF-β1, 0–1 vs. 2–3) from plasma and proportion of immune cells in malignant body fluid had a significant prognostic role in mGC. **a** Survival analysis according to the number of high levels of cytokines did not meet the statistical significance for overall survival (*P* = 0.1). **b** Patients with high levels of at least two cytokines showed significantly shorter OS than patients with zero or one cytokine at high level (median OS, 1.6 vs. 2.2 months, *P* = 0.032, **c-e** Patients with high proportion of CD8 T lymphocyte with memory marker (CD8CD45RO) and late activation marker (CD8HLA-DR) and CD3 T lymphocyte with PD-1 (CD3PD1) were associated with poor prognosis. **f** Patients with high number of FoxP3+ cells were significantly associated with favorable prognosis than mGC with low number of FoxP3+ cells in body fluid. mGC, metastatic gastric cancer. Kaplan Meier survival analysis was performed for overall survival

In survival analysis based on cytokines, high level of each cytokine in body fluid (VEGF-A, IL-10, or TGF-β1) showed poor survival outcome with borderline tendency (**Data not shown**). However, patients with high levels of at least two cytokines showed significantly shorter OS than patients with zero or one cytokine at high level (median OS, 1.6 vs. 2.2 months, *P* = 0.032, Fig. 3a-b).

Survival analysis was also conducted using immune cell profiles. Results are shown in Fig. 3c-f. Higher proportions of CD4/CD8 ratio, memory T cells (CD3CD45RO, CD4CD45RO, CD8CD45RO), and T lymphocytes with activation marker (CD3HLA-DR, CD4HLA-DR, CD8HLA-DR, CD4CD25) or inhibitory marker (CD3PD1, CD8PD1) were significantly associated with poor prognosis in univariate analysis (Table 2 and Additional file 3: Table S3). In multivariate analysis, proportion of CD8 T lymphocyte with memory marker (CD8CD45RO) and late activation marker (CD8HLA-DR), CD3 T lymphocyte with PD-1 (CD3PD1), number of FoxP3+ Tregs, and previous palliative chemotherapy history remained as independent prognostic factors **(**Table 2**)**.

**Table 2:** Univariate analysis for overall survival

		Univariate analysis				Multivariate analysis			
		HR	95% CI		*P*	HR	95% CI		*P*
Age (years)	< 58 years	1			0.913				
	≥58 years	1.03	0.57	1.86					
Sex	Male	1			0.831				
	Female	0.94	0.51	1.73					
Disease presentation	Recurrent	1			0.327				
	Metastatic	1.42	0.70	2.89					
Differentiation	WD	1			0.602				
	MD	4.94	0.61	39.76					
	PD	4.41	0.59	32.82					
	SRC	3.41	0.44	26.72					
	Others*	3.64	0.38	35.20					
HER2	Negative	1			0.892				
	Positive	0.81	0.34	1.93					
Palliative chemotherapy history	No	1			0.003	1			0.005
	Yes	4.19	1.63	10.78		10.78	2.05	56.81	
Molecular subtype of primary tumor	MSI	0.87	0.11	6.64	0.143				
	EBV	19.11	1.90	192.25					
	CIN-like	1.01	0.49	2.07					
	GS-like	1							
	Unknown	1.33	0.63	2.84					
VEGF-A (pg/mL)	< 100.9 pg/mL	1			0.127				
	≥100.9 pg/mL	1.58	0.88	2.86					
IL-10 (pg/mL)	> 243.8 pg/mL	1			0.16				
	≥243.8 pg/mL	1.64	0.82	3.25					
TGF-β1 (pg/mL)	< 393.6 pg/mL	1			0.197				
	≥393.6 pg/mL	1.97	0.70	5.55					
Cytokine-scoring model	0–1	1			0.037				
(number of high level of cytokines)	2–3	1.91	1.04	3.49					
CD4/CD3 (%)	< 41.7%	1			0.232				
	≥41.7%	0.65	0.32	1.32					
CD8/CD3 (%)	< 33.3%	1			0.272				
	≥33.3%	1.58	0.70	3.57					
CD4/CD8 ratio	< 0.6	1			0.034				
	≥0.6	0.35	0.13	0.92					
CD8CD45RO+/CD8 (%)	< 67.2%	1			0.021	1			0.019
	≥67.2%	2.54	1.15	5.59		3.25	1.22	8.68	
CD8CD25+/CD8 (%)	< 1.6%	1			0.081				
	≥1.6%	1.92	0.92	3.99					
CD8CD69+/CD8 (%)	< 28.0%	1			0.054				
	≥28.0%	2.09	0.99	4.41					
CD8HLA-DR+/CD8 (%)	< 28.4%	1			0.008	1			0.012
	≥28.4%	3.77	1.43	9.96		5.36	1.45	19.88	
CD8 TIM3+/CD8 (%)	< 0.2%	1			0.082				
	≥0.2%	0.50	0.23	1.09					
CD8 LAG3+/CD8 (%)	< 3.7%	1			0.098				
	≥3.7%	1.92	0.89	4.17					
CD8 PD1+/CD8 (%)	< 50.2%	1			0.038				
	≥50.2%	2.58	1.06	6.31					
CD3 PD1+/CD3 (%)	< 17.2%	1			0.001	1			< 0.001
	≥17.2%	3.53	1.66	7.52		8.61	2.81	26.37	
CD4 PD1+/CD4 (%)	< 32.1%	1			0.052				
	≥32.1%	2.11	0.99	4.50					
FoxP3+ Tregs (No)	< 380	1			0.029	1			0.001
	≥380	0.40	0.18	0.91		0.13	0.04	0.42	
PD-L1 positive tumor cells (%)	< 1%	1			0.87				
	≥1%	1.07	0.49	2.34					

*VEGF* vascular endothelial growth factor, *IL* interleukin, *TGF-β1* transforming growth factor- beta1, *CD* cluster of differentiation, *WD* well differentiated, *MD* moderate differentiated, *PD* poorly differentiated, *SRC* signet ring cell carcinoma, *MSI* microsatellite instability, *EBV* Epstein-Barr virus, *CIN* chromosomal instability, *GS* genomically stable, *HR* hazard ratio, *CI* confidence interval, *P* p-value, *No* number

Others*: mucinous adenocarcinoma, undifferentiated carcinoma

### Landscape analysis according to immune profile of body fluid in each molecular subtype

**Fig. 4:**
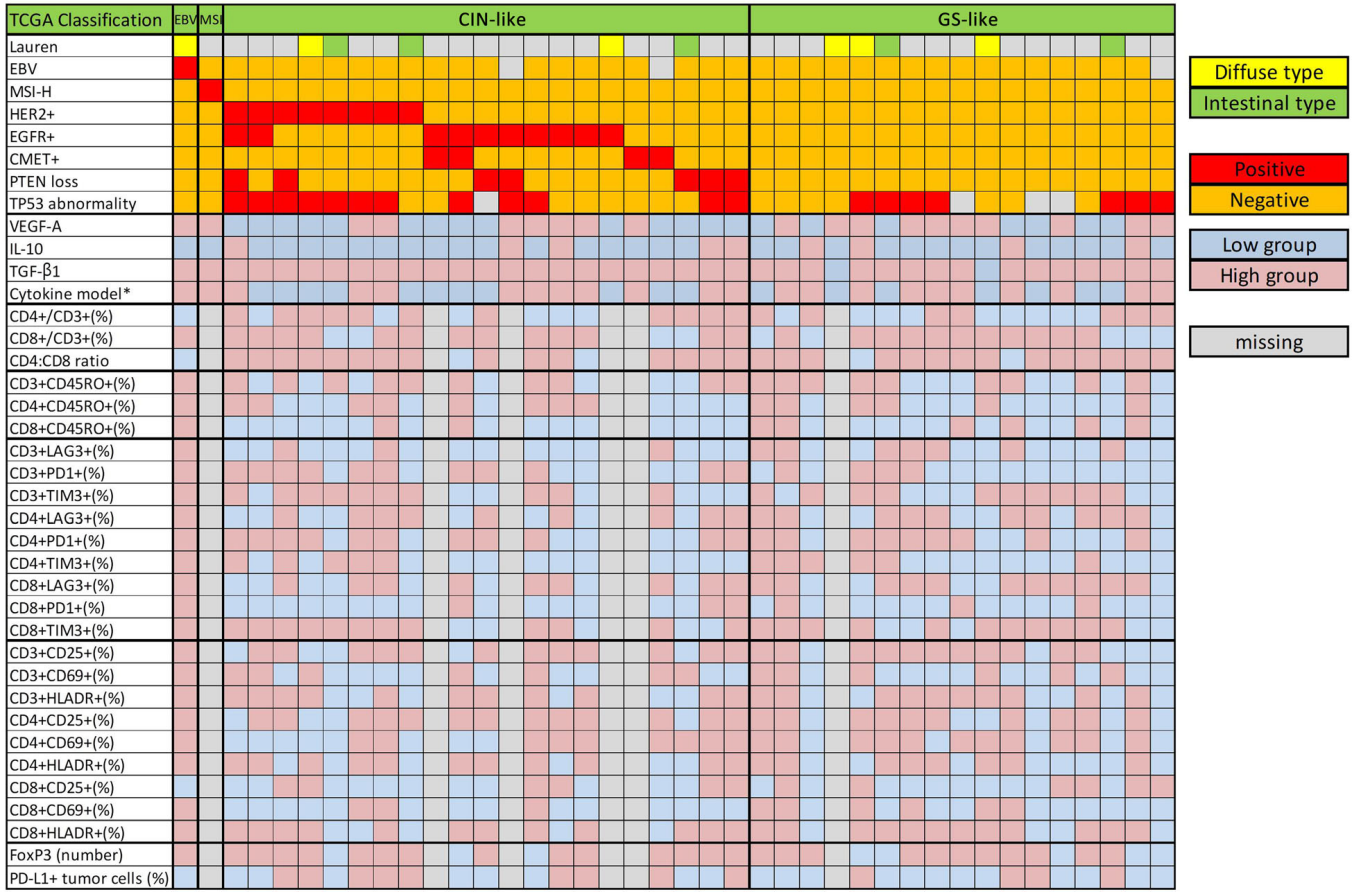
Landscape shows the immune signature of body fluid in each molecular subtype of primary tissue. Patients were categorized by The Cancer Genome Atlas (TCGA) molecular subtype which was composed of Epstein Barr Virus (EBV) positive, microsatellite instability (MSI), genomically stable (GS)-like and chromosomal instability (CIN)-like. Diffuse type and intestinal type by Lauren classification were filled with yellow and green, respectively. Molecular markers by histochemistry were classified with positive (red) or negative (orange). Continuous value of cytokine and immune cell proportion were dichotomized by best-cut off which calculates the maximizing hazard ratio (HR) based on log-rank statistics (low vs. high). Low value was filled with blue while high value was filled with pink. * Cytokine model was scored by a number of high levels of VEGF-A, IL-10 and TGF-β1, and it was categorized 0–1 (low group) and 2–3 (high group). TCGA, The Cancer Genome Atlas; EBV, Epstein-Barr virus; MSI-H, microsatellite instability-high; CIN, chromosomal instability; GS, genomically stable; VEGF, vascular endothelial growth factor; IL, interleukin; TGF-β1, transforming growth factor- beta1; CD, cluster of differentiation

**Fig. 5:**
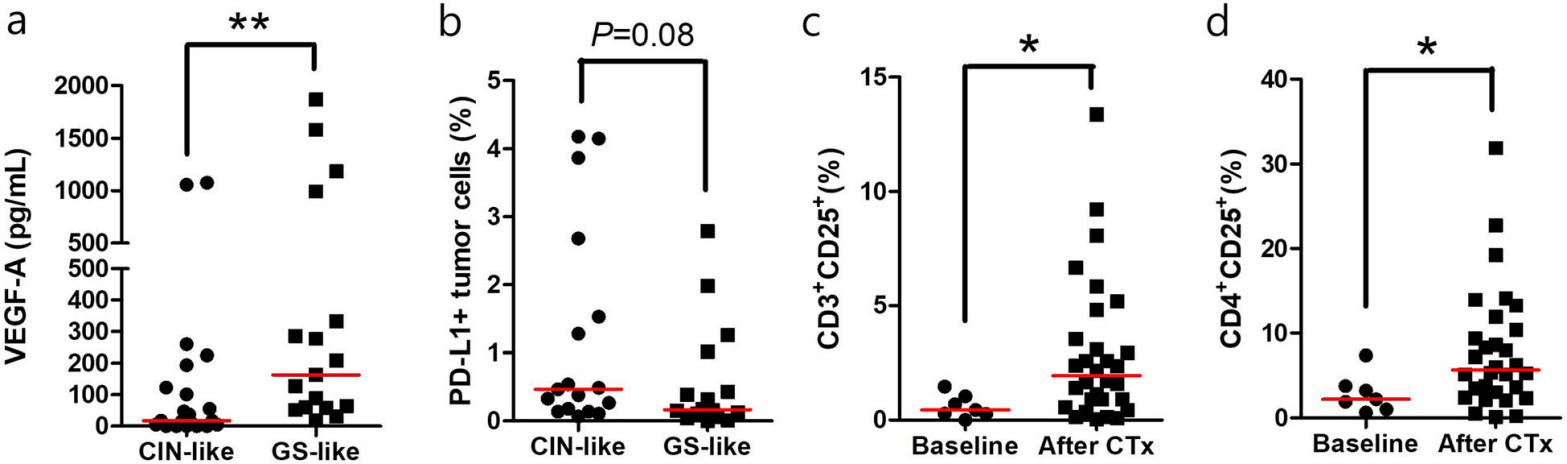
VEGF-A and PD-L1 positivity on tumor cell (%) are different between CIN-like and GS-like group, and proportion of T lymphocyte with CD25 is higher in previously chemotherapy treated group than chemotherapy naïve group. **a** VEGF-A level was significantly higher in GS-like group than that in CIN-like group (median value: 163.9 vs. 17.4 pg/mL, *P* = 0.003). **b** PD-L1 (%) of tumor cells showed a higher tendency in CIN-like group than that in GS-like group (median proportion: 0.47 vs. 0.17%, *P* = 0.08). **c-d** Proportion of early activated T cells (CD3CD25 and CD4CD25) in previously chemotherapy treated patients was higher level than that in chemotherapy naive patients (*P* = 0.017 and *P* = 0.035, respectively). Red line indicates median value. Mann-Whitney U test was used for statistical analysis. *P < 0.05, **P < 0.01

Patients were classified by molecular subgroups of primary tumor. Patterns of cytokine and immune cell profile from body fluid were compared with molecular subgroups (Fig. 4). Only 1 patient was included in EBV positive and MSI group, respectively. Thus, we focused on CIN-like group (defined as those with overexpression of RTKs) and GS-like group (defined as those who were negative for all markers including EBV, microsatellite, and RTKs) to evaluate differences in cytokine and immune profiling. VEGF-A level was significantly higher in GS-like group than that in CIN-like group (median value: 163.9 vs. 17.4 pg/mL, *P* = 0.003). No significant difference was observed in immune cell profile, although percentage of PD-L1 positive tumor cells showed a higher tendency in CIN-like group than that in GS-like group (median proportion: 0.47 vs. 0.17%, *P* = 0.08) (Fig. 5a-b).

### Dynamics of immune profile by chemotherapy

Cytokine and immune cell profile from body fluid were compared between chemotherapy naïve and previously treated groups. Proportion of early activated T cells (CD3CD25 and CD4CD25) in previously chemotherapy treated patients was higher level than that in chemotherapy naive patients (*P* = 0.017 and *P* = 0.035, respectively, Fig. 5c-d). Fraction of memory T cell with CD45RO showed higher tendency in chemotherapy treated patients than that in chemotherapy naïve patients (CD3CD45, *P* = 0.084; CD8CD45, *P* = 0.157). In addition, body fluid of patients treated with chemotherapy had higher VEGF-A levels than chemotherapy naïve patients (median value, 59.7 vs. 31.4 pg/mL, *P* = 0.535), suggesting that chemotherapy might induce immune suppressive environment.

## Discussion

Malignant body fluid of mGC has diverse cytokines and immune cells which can represent tumor microenvironment. It is relatively easy to access. In this study, immune suppressive cytokines of malignant ascites were increased compared to those of non-cancerous ascites. These cytokines are significantly associated with diverse subsets of immune cells. Immune cells with CD8CD45RO, CD8HLA-DR, CD3PD1, and FoxP3+ Tregs had a prognostic role in mGC. In addition, cytokine and immune cell profiles of body fluid were different according to molecular subtype of primary tumor and they can be changed by cytotoxic chemotherapy.

As an extension of this study, in real-world practice, angiogenesis inhibitor such as ramucirumab which had inhibitory mechanism of interaction between VEGFR2 and VEGFs [27] could be preferred for mGC with higher level of VEGF-A in malignant body fluid at any time point. In addition, ICI could be given to patients with lower level of VEGF-A (those of CIN-like group which had higher percentage of PD-L1 positive tumor cells), despite the lack of strong evidence. In the near future, biomarker studies can be performed for mGC patients who have developed malignant body fluid to evaluate the role of VEGF-A as a predictive marker for angiogenesis inhibitor or immune checkpoint inhibitor in randomized clinical trials.

Tumor secrets various immune suppressive cytokines such as VEGF, IL-6, and IL-10 to promote the accumulation of heterogeneous populations of tumor associated macrophages (TAMs), myeloid-derived suppressor cells (MDSCs), and regulatory T cells [28]. VEGF-A, IL-10, and TGF-β1 have been previously studied in metastatic ovarian cancer patients with ascites. Most of these studies showed that patients with higher levels of VEGF-A and IL10 had adverse prognosis [19, 29]. Likewise, our study showed that patients with higher levels of VEGF-A and IL-10 had poor prognostic tendency. TGF-β1 of body fluid was revealed as a poor prognostic factor in this study for the first time. In addition, the lowest level of TGF-β1 was observed in non-cancerous body fluid. This suggests that TGF- β1 also has biological role in non-cancerous body fluid. Using body fluid cytokines, we made a scoring model to predict prognosis of patients with mGC, but it should be validated by further studies.

Immune cell profiling of body fluid in mGC was also conducted and compared to that of plasma. Proportion of memory T cell with CD45RO and early activated T cell with CD69 was higher in malignant body fluid than that in plasma. This phenomenon was observed in other studies on ovarian cancer [30]. It might be related to malignant cells of body fluid that can induce immunogenicity. Proportions of suppressive T cell with PD-1 (CD3PD1, CD4PD1 and CD8PD1) and FoxP3+ Tregs were also increased in malignant body fluid. The mechanism of these phenomena could not be explained exactly. We can hypothesize that tumor cells in body fluid might induce body fluid to have an immune suppressive status while activation marker is increased by compensatory mechanism.

As a prognostic factor, higher proportions of CD8CD45RO, CD8HLA-DR, and CD3PD1 were independent poor prognostic factors. CD45RO and HLA-DR are generally regarded as activation markers while PD1 is a well-known suppressive marker [31, 32]. The number of FoxP3+ Tregs was an independent prognostic factor in our study, with higher number showing favorable prognostic factor. Many studies have reported the role of FoxP3+ Tregs [33]. However, prognostic values of these cells in cancer remain controversial. FoxP3+ Tregs are associated with short survival in the majority of solid tumors including melanomas, cervical, renal, and breast cancers. In contrast, FoxP3+ Tregs are associated with improved survival in colorectal and esophageal cancer [33]. It has been suggested that the role of FoxP3+ Tregs is influenced by tumor sites, molecular subtypes, and tumor stage, although related mechanisms are currently unknown.

Molecular subtype was classified into MSI, EBV, CIN-like, and GS-like group based on immunohistochemistry results, not genomics. Differences in cytokine and immune profiling were clustered by each group. VEGF-A level showed significant difference between CIN-like and GS-like group. VEGF-A level was higher in the GS-like group. However, other factors did not show statistically significant differences between these two groups. CIN-like group showed increased tendency of proportion of PD-L1 positive cancer cells than that in GS-like group. According to these differences, anti-VEGF therapy might give GS-like group more benefit while PD1 or PD-L1 inhibitor can be applied to CIN-like group. However, further mechanisms and clinical studies are needed to establish precision medicine based on body fluid monitoring.

The benefit of immune monitoring has already been studied and several approaches including tissue biopsy and blood sampling are ongoing [10, 34, 35]. Body fluid acquisition is more accessible than tissue. Thus, it is more useful as a source of predictive marker. In addition, it can be evaluated in real-time. To observe dynamics of cytokines and immune profile, we evaluated differences in cytokines and immune profile between chemotherapy naïve and treated patients. Early activated T cell marked with CD25 (CD3CD25, CD4CD25), memory T cell with CD45RO, and VEGF-A were higher in previously chemotherapy treated group. Immune suppressive status after chemotherapy can be assumed. By real-time monitoring of the immune environment of patients, more effective treatment strategy can be applied.

This study has some limitations. Firstly, a small number of patients were analyzed. Therefore, it is hard to have enough statistical power. Results of this study should be validated through more studies with larger sample size. Second, we only checked a limited number of cytokines and immune cell markers. This should be overcome by multiplex technology including cytometry by time of flight (CyTOF). In addition, we did not perform comparison between body fluid and tumor tissues due to invasiveness of tumor biopsy and poor performance of most patients. However, this study also has several advantages. Healthy volunteer and non-cancerous patients with ascites were included as controls to find the distinct meaning of immune profiling from malignant body fluid. In most studies, characterization and prognostic role of malignant body fluid were reported in ovarian cancer. Study of mGC body fluid was limited. We comprehensively characterized cytokines and immune profile of body fluid and evaluated the possibility of using body fluid as a monitoring source for predicting prognosis and marking therapeutic decision. In addition, we checked the proportion of PD-L1 positive tumor cells in body fluid. However, further studies with serial sampling of body fluid acquisition and immune profiling of paired primary tumor tissue are needed to verify our results.

## Additional files

Additional file 1: Cytokine level of plasma and body fluid in healthy volunteers, non-cancerous and gastric cancer patients. (DOCX 16 kb)

Additional file 2: Immune cell profiling of peripheral blood and body fluid in metastatic gastric cancer patients. (DOCX 20 kb)

Additional file 3: Univariate analysis of body fluids immune cell subset for overall survival. (DOCX 20 kb)

Additional file 4: Correlation of cytokine levels between plasma and body fluid. (PPTX 80 kb)

Additional file 5: Correlation of different cytokines in body fluid. (PPTX 81 kb)

Additional file 6: Correlation between FoxP3+ T regulatory cells (Tregs) and immune cells with suppressive markers. (PPTX 147 kb)

Additional file 7: Correlation between VEGF-A and proportion of immune cells in the body fluid. (PPTX 190 kb)

## Data Availability

The datasets used and/or analyzed during the current study are available from the corresponding author on reasonable request.

## Abbreviations

CD: Cluster of Differentiation
CI: Confidence Interval
CIN: Chromosomal Instability
EBV: Epstein-Barr Virus
GS: Genomically Stable
HLA: Human Leukocyte Antigen
HR: Hazard Ratio
IL: Interleukin
MD: Moderate Differentiated
mGC: Metastatic Gastric Cancer
MSI: Microsatellite Instability
P: *P*-Value
PD-1: Programmed Death 1
PD-L1: Programmed Death Ligand 1
SRC: Signet Ring Cell
TCGA: The Cancer Genome Atlas
TGF- β1: Transforming Growth Factor-Beta 1
VEGF: Vascular Endothelial Growth Factor
WD: Well Differentiated
