# Supplementary material for: Comprehensive immune profiling and immune-monitoring using body fluid of patients with metastatic gastric cancer
Source: J Immunother Cancer. 2019 Oct 21;7:268. doi: 10.1186/s40425-019-0708-8 (PMC6805480; doi:10.1186/s40425-019-0708-8)
Supplement: Supplementary file 1 — Cytokine level of plasma and body fluid in healthy volunteers, non-cancerous and gastric cancer patients. (DOCX 16 kb) [file 40425_2019_708_MOESM1_ESM.docx]

**Supplementary Table S1. Cytokine level of plasma and body fluid in healthy volunteers, non-cancerous and gastric cancer patients**

|  | Plasma | | | Body fluid | |
| --- | --- | --- | --- | --- | --- |
| Median (range) | Healthy volunteers (n=15) | Non-cancerous patients (n=4) | Gastric cancer patients (n=24) | Non-cancerous patients (n=11) | Gastric cancer patients (n=55) |
| VEGF-A (pg/mL) | 0 (0-0) | 0 (0-0) | 0 (0-41.9) | 0 (0-133.7) | 55.2 (0-1,865) |
| IL-10 (pg/mL) | 0 (0-0.1) | 0 (0-4.4) | 1.8 (0-44.8) | 38.0 (0-149.4) | 97.7 (0-649.2) |
| TGF-β1 (pg/mL) | 1,365 (412.4-1,774) | 1,691 (1,358-2,120) | 1356 (384.4-2,552) | 264.5 (27.6-838.1) | 1,104 (123.6-2,928) |

VEGF, vascular endothelial growth factor; IL, interleukin; TGF-β1, transforming growth factor- beta1; n, number
