# Supplementary material for: Comprehensive immune profiling and immune-monitoring using body fluid of patients with metastatic gastric cancer
Source: J Immunother Cancer. 2019 Oct 21;7:268. doi: 10.1186/s40425-019-0708-8 (PMC6805480; doi:10.1186/s40425-019-0708-8)
Supplement: Supplementary file 2 — Immune cell profiling of peripheral blood and body fluid in metastatic gastric cancer patients. (DOCX 20 kb) [file 40425_2019_708_MOESM2_ESM.docx]

**Supplementary Table S2. Immune cell profiling of peripheral blood and body fluid in metastatic gastric cancer patients**

| median (range) | Peripheral blood | Body fluids | *P* |
| --- | --- | --- | --- |
| CD4/CD3 (%) | 45.6 (30.3-65.7) | 42.8 (21.5-80.8) | 0.126 |
| CD8/CD3 (%) | 34.2 (20.9-53.7) | 42.1 (19.7-57.6) | 0.073 |
| CD4/CD8 ratio | 1.5 (0.6-2.8) | 1.1 (0.4-4) | 0.075 |
| CD3CD45RO/CD3 (%) | 43.6 (16.2-58.9) | 64.9 (30.5-97.6) | <0.001 |
| CD4CD45RO/CD4 (%) | 53.4 (18.0-76.5) | 82.0 (22-99.1) | <0.001 |
| CD8CD45RO/CD8 (%) | 30.8 (9.96-57.7) | 54.5 (20.6-93.5) | <0.001 |
| FoxP3+ (No) | 331 (17-768) | 535.5 (27.0-2,969) | <0.001 |
| PD-L1 positive  tumor cells (%) | Not measured | 0.4 (0.00-4.2) |  |
| CD3CD25/CD3 (%) | 0.9 (0-9.0) | 1.5 (0.01-13.4) | 0.298 |
| CD4CD25/CD4 (%) | 5.9 (0-20.4) | 5.2 (0.1-31.9) | 0.954 |
| CD8CD25/CD8 (%) | 0.6 (0-7.0) | 0.6 (0-17.3) | 1 |
| CD3CD69/CD3 (%) | 0.6 (0-9.8) | 5.9 (0.1-44.7) | <0.001 |
| CD4CD69/CD4 (%) | 0.6 (0-9.5) | 6.5 (0.7-52.5) | <0.001 |
| CD8CD69/CD8 (%) | 4.2 (0-16.3) | 21.1 (3.5-67.7) | <0.001 |
| CD3HLA-DR/CD3 (%) | 21.5 (5.3-48.8) | 27.3 (4.4-63.8) | 0.157 |
| CD4HLA-DR/CD4 (%) | 8.0 (2.9-36.4) | 20 (2.3-61.1) | 0.006 |
| CD8HLA-DR/CD8 (%) | 36.0 (5.0-70.3) | 39.1 (4.1-80.8) | 0.6 |
| CD3 LAG3/CD3 (%) | 1.4 (0-22.2) | 2.2 (0-23.9) | 0.723 |
| CD4 LAG3/CD4 (%) | 1.1 (0-18.6) | 6.2 (0-28.0) | 0.334 |
| CD8 LAG3/CD8 (%) | 1.3 (0-22.6) | 5.1 (0-21.1) | 0.058 |
| CD3 PD1/CD3 (%) | 5.9 (0.2-20.4) | 15.4 (0.2-51.0) | <0.001 |
| CD4 PD1/CD4 (%) | 9.7 (0.01-53.3) | 34.3 (1.3-75.6) | <0.001 |
| CD8 PD1/CD8 (%) | 17.0 (1.2-41.6) | 38.3 (0.2-74.2) | <0.001 |
| CD3 TIM3/CD3 (%) | 0.8 (0-5.3) | 1.1 (0-7.8) | 0.447 |
| CD4 TIM3/CD4 (%) | 0.2 (0-7.4) | 0.7 (0-12.6) | 0.194 |
| CD8 TIM3/CD8 (%) | 0.9 (0-9.5) | 0.8 (0-6.1) | 0.351 |

CD, cluster of differentiation; No, number; P, p-value
