# Supplementary material for: Comprehensive immune profiling and immune-monitoring using body fluid of patients with metastatic gastric cancer
Source: J Immunother Cancer. 2019 Oct 21;7:268. doi: 10.1186/s40425-019-0708-8 (PMC6805480; doi:10.1186/s40425-019-0708-8)
Supplement: Supplementary file 3 — Univariate analysis of body fluids immune cell subset for overall survival. (DOCX 20 kb) [file 40425_2019_708_MOESM3_ESM.docx]

**Supplementary Table S3. Univariate analysis of body fluids immune cell subset for overall survival**

|  |  | No | HR | 95% CI | | *P* |
| --- | --- | --- | --- | --- | --- | --- |
| CD3CD45RO+/CD3 (%) | <62.5% | 16 | 1 |  |  | 0.027 |
|  | ≥62.5% | 21 | 2.35 | 1.10 | 5.00 |  |
| CD4CD45RO+/CD4 (%) | <85.6% | 22 | 1 |  |  | 0.006 |
|  | ≥85.6% | 15 | 2.78 | 1.34 | 5.76 |  |
| CD3CD25+/CD3 (%) | <0.6% | 10 | 1 |  |  | 0.117 |
|  | ≥0.6% | 27 | 2.05 | 0.84 | 5.05 |  |
| CD4CD25+/CD4 (%) | <2.4% | 10 | 1 |  |  | 0.022 |
|  | ≥2.4% | 27 | 2.87 | 1.16 | 7.09 |  |
| CD3CD69+/CD3 (%) | <7.2% | 22 | 1 |  |  | 0.25 |
|  | ≥7.2% | 15 | 0.64 | 0.29 | 1.38 |  |
| CD4CD69+/CD4 (%) | <4% | 13 | 1 |  |  | 0.106 |
|  | ≥4% | 24 | 1.96 | 0.87 | 4.41 |  |
| CD3-HLA-DR+/CD3 (%) | <21.8% | 13 | 1 |  |  | 0.002 |
|  | ≥21.8% | 24 | 3.84 | 1.61 | 9.17 |  |
| CD4HLA-DR+/CD4 (%) | <17.9% | 16 | 1 |  |  | 0.011 |
|  | ≥17.9% | 21 | 2.73 | 1.26 | 5.92 |  |
| CD3 LAG3+/CD3 (%) | <6.4% | 24 | 1 |  |  | 0.333 |
|  | ≥6.4% | 13 | 1.45 | 0.69 | 3.06 |  |
| CD4 LAG3+/CD4 (%) | <4.3% | 15 | 1 |  |  | 0.371 |
|  | ≥4.3% | 22 | 1.40 | 0.67 | 2.93 |  |
| CD3 TIM3+/CD3 (%) | <0.7% | 13 | 1 |  |  | 0.053 |
|  | ≥0.7% | 24 | 0.47 | 0.22 | 1.01 |  |
| CD4 TIM3+/CD4 (%) | <1.2% | 24 | 1 |  |  | 0.089 |
|  | ≥1.2% | 13 | 1.91 | 0.91 | 4.05 |  |

CD, cluster of differentiation; No, number; HR, hazard ratio; CI, confidence interval; P, p-value
