# Supplementary figures and images for: Comprehensive immune profiling and immune-monitoring using body fluid of patients with metastatic gastric cancer
Source: J Immunother Cancer. 2019 Oct 21;7:268. doi: 10.1186/s40425-019-0708-8 (PMC6805480; doi:10.1186/s40425-019-0708-8)

## Slide 1
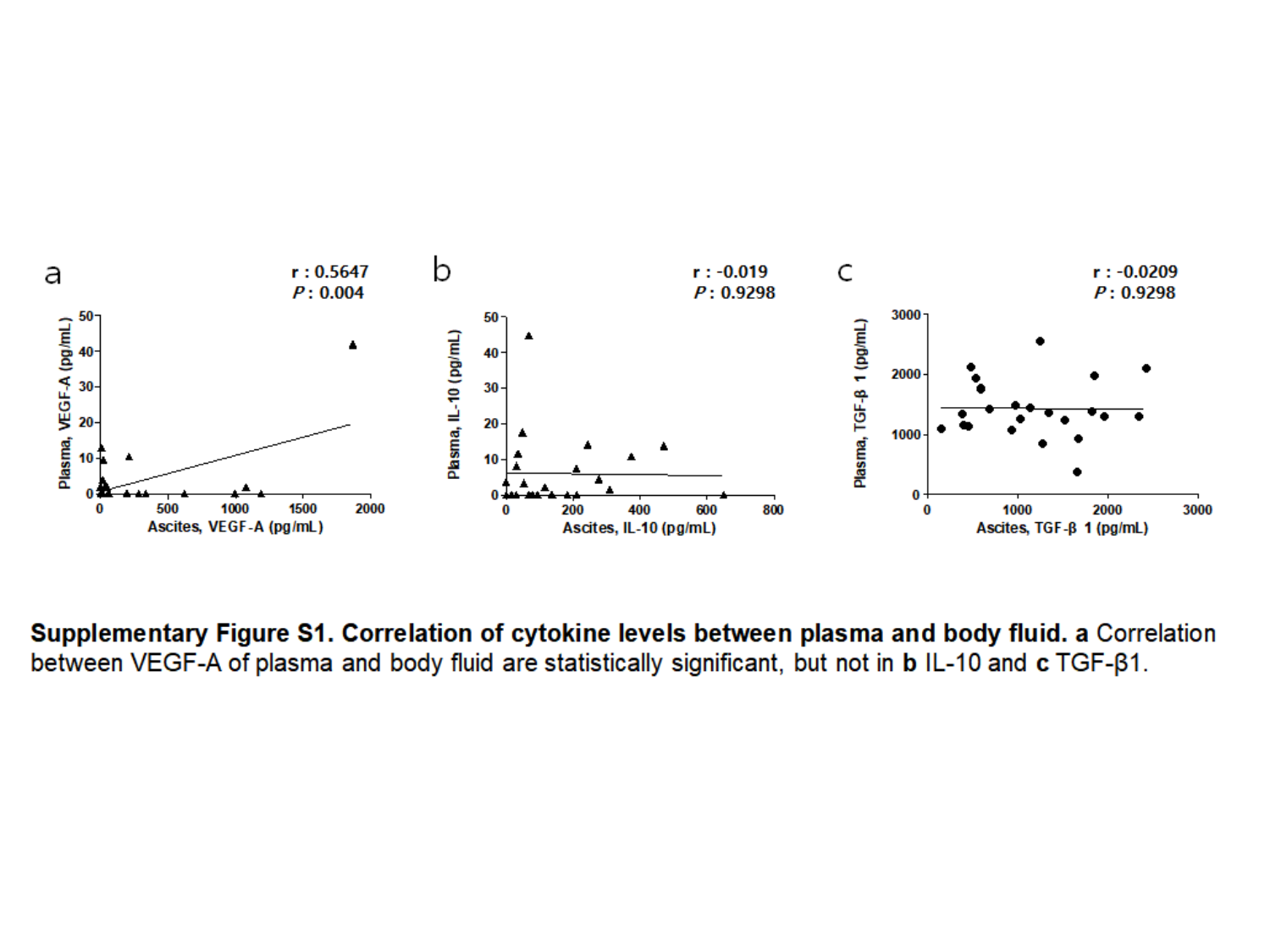

Supplement: Supplementary file 4 — Correlation of cytokine levels between plasma and body fluid. (PPTX 80 kb) [file 40425_2019_708_MOESM4_ESM.pptx]

## Slide 1
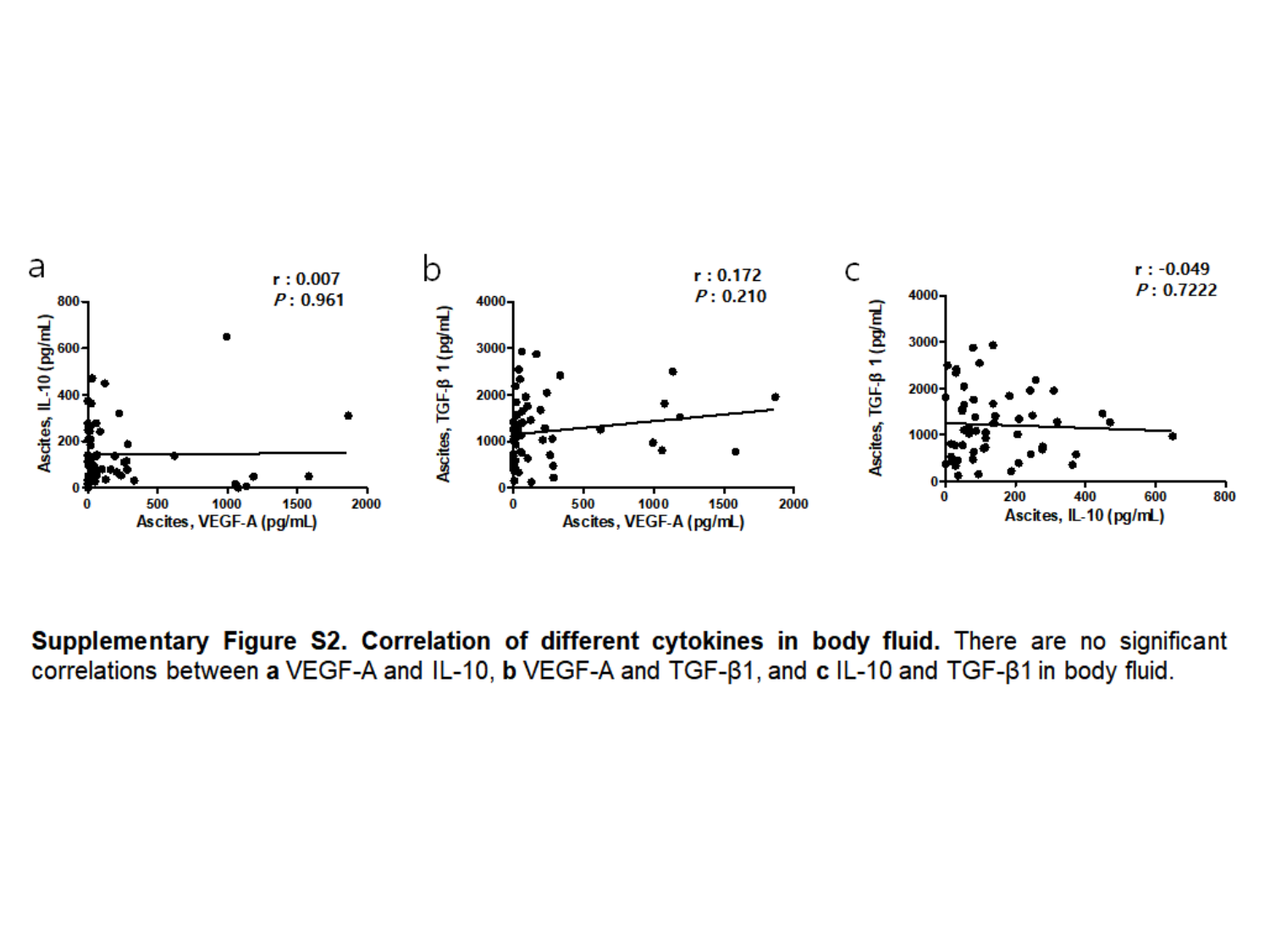

Supplement: Supplementary file 5 — Correlation of different cytokines in body fluid. (PPTX 81 kb) [file 40425_2019_708_MOESM5_ESM.pptx]

## Slide 1
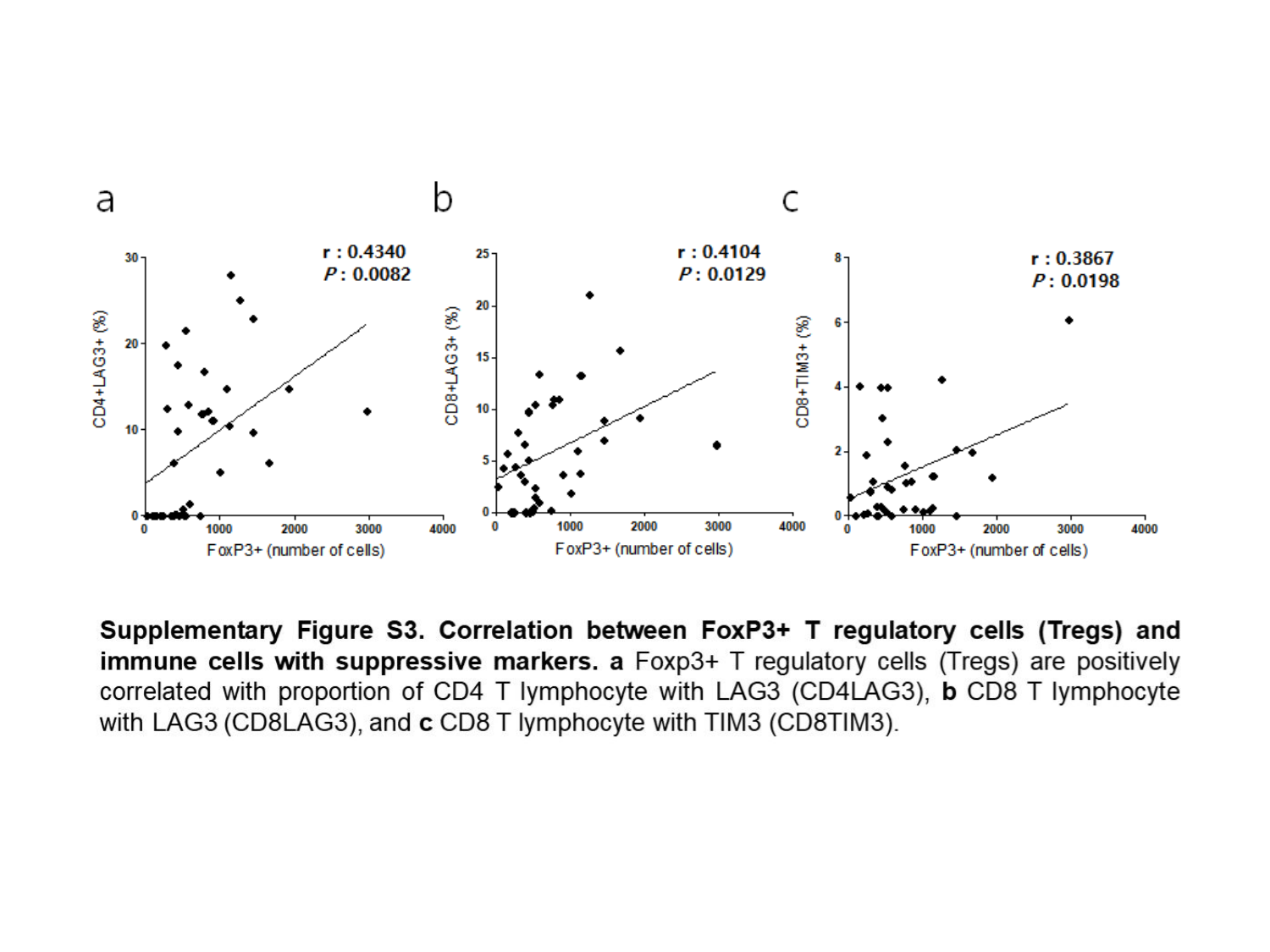

Supplement: Supplementary file 6 — Correlation between FoxP3+ T regulatory cells (Tregs) and immune cells with suppressive markers. (PPTX 147 kb) [file 40425_2019_708_MOESM6_ESM.pptx]

## Slide 1
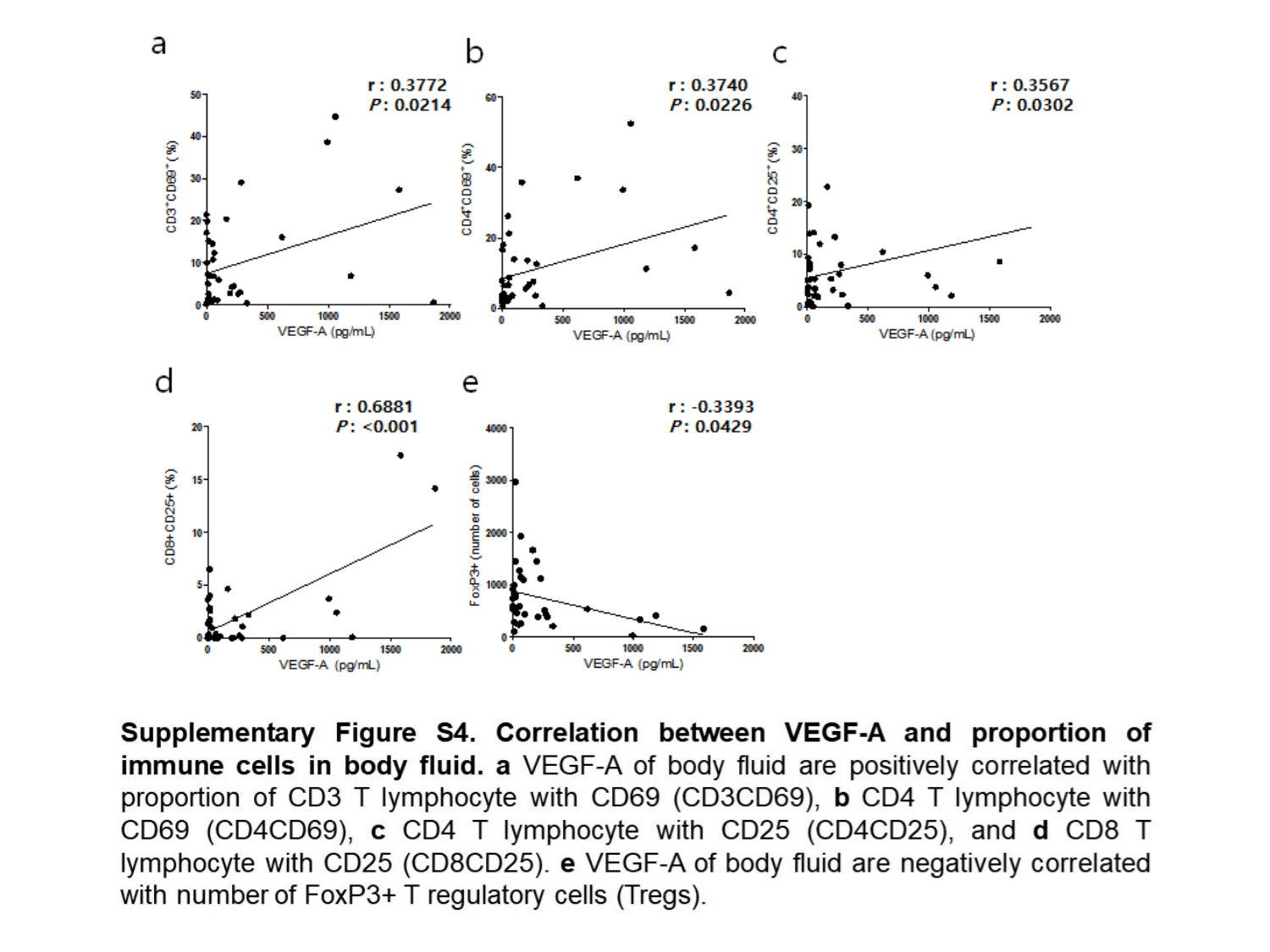

Supplement: Supplementary file 7 — Correlation between VEGF-A and proportion of immune cells in the body fluid. (PPTX 190 kb) [file 40425_2019_708_MOESM7_ESM.pptx]
